# Barriers to and strategies to address COVID-19 testing hesitancy: a rapid scoping review

**DOI:** 10.1186/s12889-022-13127-7

**Published:** 2022-04-14

**Authors:** Mark Embrett, S. Meaghan Sim, Hilary A. T. Caldwell, Leah Boulos, Ziwa Yu, Gina Agarwal, Rhiannon Cooper, Allyson J. Gallant AJ, Iwona A. Bielska, Jawad Chishtie, Kathryn Stone, Janet Curran, Andrea Tricco

**Affiliations:** 1 Faculty of Health, Dalhousie University, Halifax, NS Canada; 2 Research, Innovation & Discovery, Nova Scotia Health Authority, Halifax, NS Canada; 3 Healthy Populations Institute, Dalhousie University, Halifax, NS Canada; 4 Maritime SPOR Support Unit, Halifax, NS Canada; 5 School of Nursing, Dalhousie University, Halifax, NS Canada; 6 Department of Family Medicine, McMaster University, Hamilton, ON Canada; 7 Department of Health Research Methods, Evidence and Impact, McMaster University, Hamilton, ON Canada; 8 Health Services Outcomes and Evaluation Unit, Faculty of Medicine, Rehabilitation Sciences Institute, University of Toronto, Toronto, Canada; 9 School of Health and Human Performance, Dalhousie University, Halifax, NS Canada; 10 IWK Health Centre, Halifax, NS Canada; 11 Knowledge Translation Program, Li Ka Shing Knowledge Institute, St. Michael’s Hospital, Toronto, Canada; 12 Epidemiology Division, Dalla Lana School of Public Health and Institute for Health, Management, and Evaluation, University of Toronto, Toronto, Canada

**Keywords:** Covid-19, Testing, Testing hesitancy, Health policy, Social determinants of health, Equity, Coronavirus, Scoping review

## Abstract

**Background:**

Testing is a foundational component of any COVID-19 management strategy; however, emerging evidence suggests that barriers and hesitancy to COVID-19 testing may affect uptake or participation and often these are multiple and intersecting factors that may vary across population groups. To this end, Health Canada’s COVID-19 Testing and Screening Expert Advisory Panel commissioned this rapid review in January 2021 to explore the available evidence in this area. The aim of this rapid review was to identify barriers to COVID-19 testing and strategies used to mitigate these barriers.

**Methods:**

Searches (completed January 8, 2021) were conducted in MEDLINE, Scopus, medRxiv/bioRxiv, Cochrane and online grey literature sources to identify publications that described barriers and strategies related to COVID-19 testing.

**Results:**

From 1294 academic and 97 grey literature search results, 31 academic and 31 grey literature sources were included. Data were extracted from the relevant papers. The most cited barriers were cost of testing; low health literacy; low trust in the healthcare system; availability and accessibility of testing sites; and stigma and consequences of testing positive. Strategies to mitigate barriers to COVID-19 testing included: free testing; promoting awareness of importance to testing; presenting various testing options and types of testing centres (i.e., drive-thru, walk-up, home testing); providing transportation to testing centres; and offering support for self-isolation (e.g., salary support or housing).

**Conclusion:**

Various barriers to COVID-19 testing and strategies for mitigating these barriers were identified. Further research to test the efficacy of these strategies is needed to better support testing for COVID-19 by addressing testing hesitancy as part of the broader COVID-19 public health response.

**Supplementary Information:**

The online version contains supplementary material available at 10.1186/s12889-022-13127-7.

## Background

COVID-19 is a disease caused by the SARS-CoV-2 virus that was first discovered in December 2019 and declared a global pandemic on March 11, 2020, [1]. Since then, there have been over 170 million cases worldwide [2]. COVID-19 is confirmed by testing, which plays an important role in the find-test-trace-isolate-support cycle to control the spread of COVID-19. Find-test-trace-isolate-support describes testing anyone with symptoms of COVID-19, tracing their contracts, and isolating these individuals until they are no longer contagious [3]. While this process can prevent widespread outbreaks of the disease, there are other consequences of widespread testing such as separation from family members while waiting for test results.

In response to the rapidly growing body of evidence about COVID-19, and the understanding that a find-test-trace-isolate-support public health response is needed, the Canadian Minister of Health commissioned a COVID-19 Testing and Screening Expert Advisory Panel to provide evidence-informed advice to the federal government on science and policy related to testing and screening [4]. The panel consulted with over 80 health experts, public policy experts and members of industry to develop a robust testing approach across Canada. In late 2020, the authors of this review were approached to conduct a rapid scoping review of COVID-19 testing hesitancy to help the Panel develop guidance about COVID-19 testing.

### COVID-19 testing and testing hesitancy

In most jurisdictions, testing is managed by public health authorities. The gold standard for COVID-19 diagnosis is reverse transcription polymerase chain reaction (rRT-PCR) testing from a throat swab or nasopharyngeal swab [5]. Diagnosis of COVID-19 allows people and public health authorities to be aware of infections and to take actions to limit the spread to others. Once a test is confirmed positive, it is recommended that the individual and any close contacts isolate for 14 days [6]. Despite the population health benefits of COVID-19 testing, preliminary evidence shows that COVID-19 testing barriers exist along multiple and intersecting dimensions. Barriers to testing may be the result of testing centre hours, inaccessible environments, locations of testing centres, communication strategies and decisions of how testing is allocated [4]. Information about implications of a testing outcomes created barriers, such as perceived negative social and economic consequences of a positive test, such as loss of income or employment or social stigma. These barriers (informational, accessibility, social and economic) create hesitancy in individuals to get tested for COVID-19.

The Canadian COVID-19 Expert Panel on Testing and Screening recommends that all jurisdictions implement context-specific strategies to increase testing uptake, such as testing centres in disease hot spots and targeted communication strategies. As the COVID-19 pandemic continues to impact communities around the globe, empirical evidence about the barriers to testing and strategies to mitigate barriers is needed to fully implement the find-test-trace-isolate-support model.

### Conceptual approach to organizing findings

To better understand COVID-19 testing hesitancy, we reviewed evidence that aimed to understand individual reasons for hesitating (delaying) to seek healthcare when needed. The ‘three delays’ model is a prominent model that separates the decision processes that may influence hesitancy into three decision stages [7]. The first category is the decision to seek help, which include actors that influence individual decision making (I.e., sociocultural factors, distance/access, and opportunity costs). In other words, the recognition of symptoms and the decision to seek care (planning). The second related the finding and reaching an appropriate testing facility and deciding to go through the testing process (process). The process factors are influenced by availability, cost, and travel conditions. Finally, the third category relates to receiving adequate care and determining what actions may need to be taken when an outcome is determined (outcomes). Outcomes can be influenced by availability of supplies, equipment, trained personnel, patient management, and consequences of care. The model has primarily been used to understand barriers in access to care in low and middle-income countries by highlighting individual, organizational and system level barriers [8]. For present our findings, we adapted the model to organize barriers that influence individuals to seek a COVID-19 test, including knowledge about access, symptoms, etc. (planning), the characteristics of the COVID-19 test itself (process) and consequences of the COVID-19 testing results (outcomes) (Table 1). Strategies to address these barriers were organized in the same manner (Table 2) [7]. It is noteworthy that the three delays model is useful to organize factors into planning, process, and outcome factors; however, factors are not necessarily mutually exclusive.

**Table 1:** Barriers to COVID-19 testing organized by type of delay

Planning barriers	Process barriers	Outcomes barriers
•Cost of Testing •Health literacy •Misinformation •Testing criteria (changes in testing criteria) •Health status •Trust in health system	•Availability of testing sites •Waiting times (availability of human resources, testing supplies) •Infrastructure features of testing sites •Time delay in results (including laboratory capacity) •Test properties (including pain, length of test) •Test accuracy/sensitivity (false positives) •Safety of test site (chance of infection) •Trust in the process	•Stigma •Personal cost (cost of isolation, positive test results: work, cost related to care etc.) •Consequences on employment •Health consequences

**Table 2:** Strategies to address COVID-19 testing hesitancy

Planning strategies	Process Strategies	Outcomes Strategies
•Eliminate costs of testing •Incentivize testing with rewards •Promote awareness and testing locations •Scientific communication strategy aimed to improve health literacy •Targeted communication strategies aimed at vulnerable populations to improve inequities	•More variability in type of testing sites (drive thru, walk up, at home, drone delivery etc.) •Transportation support •Culturally tailored testing sites for communities •Have community champions •Informative signage	•Housing for self-isolation •Support for tracking and tracing

### Review questions

Given the importance of COVID-19 testing in the find-test-trace-isolate model, it is necessary to understand factors that prevent people from getting tested and approaches to improve testing uptake. The primary aim of this review is to describe barriers to COVID-19 testing. The secondary aim is to describe effective communication or testing strategies to aid in reducing barriers to COVID-19 testing or addressing COVID-19 testing hesitancy. The population, concept and context for this rapid scoping review is: 1) population – persons who are eligible to be tested for COVID-19; 2) concept – COVID-19 testing; and 3) context – testing in any setting.

## Methods

We developed an a priori review protocol according to the research question. We drafted the protocol according to the updated methodological guidance for scoping reviews by Peters et al., adapted for a rapid review by the research team, members of Health Canada, and our teams’ information specialist with expertise in designing searches on topics relating to health and economics [9]. A rapid review methodology was chosen due to the time constraints upon which this request was needed to inform Health Canada’s COVID-19 Expert Panel on Testing and Screening. This review is reported according to the Preferred Reporting Items for Systematic Reviews and Meta-Analyses extension for scoping reviews (PRISMA-ScR), and the search is reported according to the PRISMA extension for literature searches [9, 10].

### Search strategy

An experienced information specialist (expert librarian) designed comprehensive search strategies for the following databases: Medline, Scopus, MedRxiv, Cochrane Database of Systematic Reviews, and Google (see Appendix A for detailed information. No study registries were searched. All database searches were executed on January 8, 2021, and results were limited to January 1, 2019-January 8^th^,2021. Grey literature was retrieved using a combination of targeted website searching and a series of Google queries. Due to the context of the rapid review process, citation searching, and expert consultation were not included in our search methods.

### Screening & data extraction

Covidence, an online tool for conducting various types of reviews (www.covidence.org) was used to review the titles and abstracts for inclusion/exclusion based on the criteria described in Appendix B. Due to the rapid nature of this review, abstracts were reviewed by single reviewers. Next, articles were single screened during full text using the same inclusion/exclusion criteria. Data extraction was completed, using Covidence, and the following endpoints: [1] country; [2] purpose or aim; [3] study design (if applicable); [4] subgroups and populations of interest; [5] barriers to testing identified; [6] strategies to address barriers or testing hesitancy – either implemented or suggested; and [7] lessons learned, recommendations and outcomes of strategies implemented (if available). The complete data extraction guidelines can be found in Appendix C.

Data Synthesis.

To synthesize the descriptive results and organize them into the three categories of the three delays model, team members extracted and organized the data based on the description of each category (provided above). Team members were instructed to verify any uncertainty with team leads (ME, MS). After extraction, all team members discussed their organization, and any conflicts or uncertainty was discussed until consensus was reached. Table 1 (barriers) and Table 2 (strategies) include the extracted factors identified in the literature.

## Results

### Overview of included studies

A total of 1294 unique published articles were identified from the database search. Another 97 grey literature sources were identified. After screening, 61 sources were included for data extraction (*n* = 30 academic publications; *n* = 31 grey literature sources; PRISMA diagram in Appendix C). Most studies were from the Unites States (*n* = 36) and the remainder were from Canada (*n* = 5), the United Kingdom or other countries (*n* = 15).

Extracted data were categorized in the following two sections: 1) barriers or factors that influence COVID-19 testing and/or testing hesitancy; and 2) strategies to mitigate COVID-19 testing barriers or hesitancy. Each section is then subdivided into sections focusing on the planning, process, and outcomes using the ‘three delays’ model conceptual framework [8].

### Planning barriers

The planning stage in the *three delays model* focuses on factors that influence the decision to seek appropriate medical care once symptoms are noticed or risk of infection is severe enough. In this case, barriers that influence decisions to seek COVID-19 testing found in the literature include the cost of testing, health literacy, trust in the health system, and health status.

### Cost of testing

Cost, often associated with lack of health insurance, was identified as a barrier to seeking COVID-19 testing by eight sources [11–17]. Fernando et al. (2020) described the prohibitive cost of testing, the cost of missing work and transportation costs associated with traveling to and from testing sites as reasons why people did not seek testing. Thunström et al. (2020) found that personal financial situations did not affect an individual’s willingness to take a free test.

### Health literacy

Health literacy was cited as a barrier to COVID-19 by nine sources [11, 18–25], Health literacy may refer to limited knowledge of testing, misinformation regarding testing and COVID-19, and/or poor recognition of symptoms. According to the COVID-19 Unified Command Report (2020), low health literacy is associated with risky health behaviours, lower likelihood of seeking treatments and care, and reduced compliance with health-related instructions/guidance, including seeking COVID-19 testing. Misinformation and mixed facts regarding protective measures, transmission, and testing protocols were cited as a reason people do not seek COVID-19 testing [21, 24, 25]. In a longitudinal survey of Australian citizens (

*n* = 1369 participants), Bonner et al. (2020) found that not knowing how or where to get tested was a common barrier to testing. Different social groups may be prone to low health literacy preventing testing for various reasons. Undocumented immigrants and foreign workers are often disconnected from the healthcare system and social services, resulting in this group not seeking testing or care when needed [22].

### Trust in the health system

Low trust in the health system, reported as a lack of trust in government and medical professionals in general, was cited as a barrier to seeking COVID-19 testing by six sources [16, 26–30]. Due to the nature of the sources, there is limited validated evidence that trust in the health system impacts the likelihood that an individual will or will not seek COVID-19 testing. Distrust in the health system combined with minority status results in decreased access to testing and overall disparities in access to COVID-19 care. Histories of systemic abuse and exploitation of minorities by the medical and research communities, such as the Tuskegee syphilis experiment, were cited by two sources [26, 29] as a cause of distrust in the healthcare system.

### Health status

There were two sources that identified current health status as a barrier to testing [31, 32]. Both sources describe the challenges of encouraging individuals who feel healthy (e.g., asymptomatic) to take COVID-19 tests. Levitt (2020) indicates that refusal for testing among people with self-perceived good health status is one of the greatest barriers to controlling COVID-19; the article further describes the societal costs of healthy individuals not getting tested and suggests ways to incentivize regular testing in this group. Kernberg (2020) determined that among individuals who declined COVID-19 testing, the second most popular reason was confidence that they were not infected with the virus.

### Process barriers

The process stage in the *three delays model* focuses on factors that influence reaching of appropriate medical services, including uncertainty about the services. In this case, barriers in this delay include the availability and infrastructure of COVID-19 testing sites, including the perceived fear of the testing process.

### Availability of testing sites

Nine sources described the availability of testing sites as a process barrier for testing, both in general [11, 26, 28, 33] and in specific communities [19, 25, 28, 30, 34]. Transportation barriers and access to testing centres play a role in accessing COVID-19 testing sites. Among adults surveyed in the United States (cross-sectional, *n* = 3058 participants), 15% reported not being aware of where to get tested or did not have means to travel to get tested [25]. In New York City, clusters analysis demonstrated that a lack of availability of testing sites in lower SES communities where there were a higher proportion of positive cases and more severe cases than higher socioeconomic communities^34^. In comparison, testing sites in Texas were often located in what was described as “whiter” communities^21^ while testing was limited in Black communities, and this was attributed to the inequitable distribution of resources [34]. Further, Maxmen et al. (2020) explained that the lack of access to testing was exacerbated in rural and remote communities. Similarly, distance to testing sites increased in rural areas, resulting in a reduction of testing access. Rader and colleagues concluded that geographic barriers to testing exacerbated health inequalities in rural counties. They recommended that geographic accessibility be considered when planning the location of testing sites. Similarly, in remote communities in Australia, testing availability and timeliness were identified as barriers to testing [35].

### Infrastructure

Two articles identified physical limitations including limited mobility, blindness, low vision, difficulty hearing, communication or understanding information and sensory challenges as barriers to testing [22, 36]. Further, long wait times for testing and test results were identified as barriers to testing in five studies [25, 37–40], where Clipman et al. (2020) reported that 53% of survey respondents (*n *= 3058) in the United Stated waited eight or more days for test results, while a drive-through testing in Phoenix had a thirteen hour wait for testing. Thappa et al. (2020) highlighted the discomfort associated with waiting at a facility for results.

Discomfort, worry, and distrust were also identified as barriers to testing. Kernberg et al. (2020) found that when offered a monosymptomatic test, 17% (of 270 patients) declined, most frequently citing concern for test discomfort. Further, an Australian national survey of 1359 citizens found that pain during testing (11%) was the most common barrier (Bonner et al., 2020). Four studies reported that the safety of testing sites and worry about infection were barriers to seeking testing [41–44]. More specifically, the fears of the safety of test sites were related to physical distancing, isolation, and cleaning practices. Among racialized Americans, Egelko et al. (2020) noted that distrust in the healthcare system and the state was present due to racist treatment that had and continued to exist within them, and the ongoing role these institutions had in perpetuating health inequities. Similarly, immigrants lacking legal status were also hesitant to receive a test, as there was a fear that the government may use personal information to ensure immigration enforcement [45].

### Outcome barriers

The outcomes stage in the *three delays model* focuses on the consequences once care is received. In this case, the consequences of a positive COVID-19 test. Barriers in this delay include the stigma associated with a positive test and the personal and health costs of a positive test.

### Stigma

Social stigma was identified as a barrier to being tested for COVID-19 in three articles [24, 46, 47]. In an online survey exploring whether stigma would influence individuals’ decision to get tested, stigma was viewed as a barrier because testing may suggest that people did not follow public health recommendations (e.g., use of personal protective equipment, physical distancing) [47]. Kissam [46] reported that among racialized Californians, there may be fear of testing due to possible repercussions of testing positive, such as substandard or stigmatized healthcare. Finally, one source identified that sex workers in Africa faced stigma and discrimination, and this may have limited their access to contact tracing and COVID-19 testing [24].

### Personal and health costs of testing positive

Five articles described personal costs identified as barriers to testing across populations [22, 33, 39, 48, 49]. Four of these articles were commentaries that describe expert opinion on how personal and health costs of testing are a barrier for low income and migrant workers who are hesitant to test positive due to potential loss of work and income need to quarantine following a positive test and fears of deportation among immigrants [22, 33, 39, 49]. UNICEF provides an evidence brief that described the infodemic of negative consequences which are discouraging individuals form being tested [48].

### Planning Strategies

#### Eliminate cost of testing/ Incentivize testing

Five articles overviewed strategies to eliminate the cost of testing, rather than improving convenience of testing [32], although no evidence was provided that these strategies would be effective. Several authors [20, 37, 48, 50], mostly from the United States, proposed improving uptake by eliminating the costs of COVID-19 tests, providing incentives to taking tests, including cash benefits, especially for low income or undocumented citizens who may have been afraid to take a test due to immigration status.

### Promote awareness

Five articles included promoting awareness as a strategy to increase testing. Doyle et al., (2020) proposed a public information campaign to inform uninsured people in the US that care was available at no charge^27^. They suggested that this would help address misconceptions about the consequences of testing. Capps et al. (2020) recommended that the federal government provide funding to local testing sites to increase uptake of symptomatic individuals^28^. Earnshaw (2020) conducted a cross-sectional survey of 845 adults in the US to examine individual characteristics such as COVID-19 stigma variables (e.g., anticipated stigma and stereotypes), COVID-19 control variables (e.g., knowledge and fear), and sociodemographic characteristics to determine behaviour towards testing^49^. Page et al. (2020) suggested funding community and religious organizations to promote awareness around testing and testing sites^18^.

### Scientific communication strategy aimed at improving health literacy

Two sources suggested that scientists, academics, and other experts were able to counteract misinformation about the pandemic, testing and consequences of a positive test to help improve public trust [23, 49]. Additionally, they suggested building a multi-disciplinary network of academic, community, public, and other partners to understand and address those most impacted by structural inequities, with a focus on testing and tracing. Khaldi et al. (2020) recommended a strategy whereby government bodies provide leadership through initiatives that address misconceptions about the pandemic, testing, outcomes, and other issues that inhibit individuals from testing.

### Targeted communication strategies aimed at vulnerable populations to improve inequities

Three article included approaches that targeted communication strategies aimed at vulnerable populations. In a commentary, Thappa et al. (2020) suggested that the Indian government target specific information and education materials that reflected the unconcerned attitude many Indian residents have toward testing [40]. This would require regular updating of information and content. Similarly, in an editorial, Sotgiu et al. (2020) recommended that the government provide a consistent stream of communication that addresses misinformation and provided honest, direct, simple communication from leaders about testing [51].

Egelko et al. (2020) proposed that unidirectional messaging was ineffective and would potentially turn individuals away. Instead, they suggested that community trust needed to be fostered with the aim of making testing an attractive option for all individuals through targeted approaches [49]. Specifically, the authors suggested framing testing as a surveillance method which allows for anonymity instead of as a case-finding method which could be infringing.

### Multifaceted strategies

The community council of Tower Hamlet in the U.K. designed a protocol that addressed a local outbreak of COVID-19 [52], including strategies for planning, process, and outcomes. For planning, they designed a survey and community mapping exercise to gather in-depth insight on support needs. Additionally, a Community Engagement Sub-group had been established to support residents taking part in the national test and trace programme. Initial findings were that community members were more likely to engage in the plan if they were approached by trusted community faith leaders. This helped build trust to convey messages regarding the benefits of testing and contact tracing and responding to data about hotspots or areas of low uptake.

### Process strategies

#### Availability of testing sites

Four articles suggested ways to improve the availability of testing sites [19, 30, 53, 54]. Murphy et al., (2020) reported on the implementation of a free testing initiative for Southeastern Pennsylvania Transportation Authority employees offered by the Black Doctors COVID-19 Consortium, to increase testing for frontline non-hospital workers. Murphy (2021) reported on the same Consortium, where female black doctors provided free tests to underserved communities. The COVID-19 Testing and Contact Tracing Health Equity Guidebook suggested addressing barriers such as transportation by using a mixed approach, including drive-through sites, walk-up sites, mobile screening, and door-to-door screening. Similarly, Maxmen et al., (2020) described a multi-pronged approach to augment access to testing, including testing at physical clinics, youth shelters and by deploying a street medicine team (using a mobile RV)—which was indicated to be useful for contact tracing among individuals experiencing unstable housing.

Adeniji et al., (2020) suggested allowing self-administered tests and education on how to use them and two others proposed establishing test sites in pharmacies [55, 56]. Another article described an initiative that used drones to deliver testing kits to remote communities, such as Stoney Nakoda First Nations, Eden Valley, and Big Horn (satellite reserves) [57]. Kissam et al., (2020) argued that COVID-19 testing in local areas should shift towards being a component of a comprehensive public health strategy along with contact tracing for those who test positive and additional supports for those required to quarantine/self-isolate.

### Accessibility

The COVID-19 Testing and Contact Tracing Health Equity Guidebook proposed offering bilingual, culturally tailored testing and contact tracing services in communities with elevated risk. Authors suggested the consideration of processes that include individuals who lack permanent contact information or have unstable housing and using recognizable locations that are familiar to the community. Maxmen et al., (2020) proposed multiple, accessible testing facilities with providers that reflect community characteristics. Similarly, the Minnesota Department of Health proposed improving accessibility of testing sites for those with physical disabilities by providing signage that is clear, visible, and easy to understand. One article recommended that trusted community leaders and organizations help develop and coordinate testing strategies [58]. Similarly, Mitchell et al., (2020) emphasized the need for collaboration and coalitions between academics, laboratories, public health, and local healthcare to target vulnerable populations and increase access to tests. Another proposed making testing sites more convenient by providing drive-through testing, which could help to address the fear of infection [59]. Galaviz et al. (2020) proposed ongoing monitoring and evaluation of testing interventions and their impact on access, where factors such as culture, history, values and needs of minority communities are considered [29].

A media article described how Oregon Health & Science University (OHSU) allotted the first two hours of the testing day for first responders, OHSU health patients, and household members of OHSU health employees [60]. To help people understand the process of testing, two healthcare professionals in Saskatchewan agreed to take a COVID-19 test despite being asymptomatic, then share their lived experience with their patients to decrease stigma and increase education about the discomfort of the test [61].

### Outcome Strategies

#### Accommodations for self-isolation & Support for tracking and tracing

One article proposed various strategies to increase testing among minority populations such as increasing the availability of testing sites in minority neighbourhoods and providing cost-free temporary accommodations for self-isolation [62]^65^. Tower Hamlets Council in the United Kingdom linked those isolating to existing supports in the community, such as faith and mutual aid groups and used locally trained and embedded volunteers to support individual residents at every stage of the national test and trace process.

## Discussion

The transmission and containment of a pandemic is, in part, determined by government policy, health system initiatives, and the active engagement of the public. To be successful, testing, an essential tool in stopping the further spread of a contagion, requires engaging of all three of these stakeholder groups. Rather than imposing strict and ineffective policies, it will be important for health system leaders to first consider the strategies that address barriers directly and have supporting evidence as to their effectiveness. Two strategies that receive a lot of support in the findings, and are feasible policy options, include: raising public awareness through promotions, and decentralizing testing centers.

A major strategic focus is to improve communication strategies for testing through public awareness campaigns about the benefits of testing and to overcome misinformation and stigma while also improving health literacy by improving [63] people access to health information and their ability to navigate the health system. Increasing awareness may also help address the changing public perceptions and acceptance of COVID-19 testing. Public health campaigns to raise awareness has been successful to increase testing for other communicable disease [64]. To overcome COVID-19 testing hesitation and increase uptake, policies and information campaigns that emphasise the significance of timely testing and rapid communication answers to enquiries and rumours, as well as create a supportive atmosphere for accessing tests, are critical. Increase awareness will provide opportunities to educate public and increase health literacy. Scientists, academics, influencers, and other experts are needed to counteract misinformation about testing, and to promote population long-term compliance.

A frequently identified planning strategy focused on improving the accessibility of testing centres for people with disabilities and multisectoral collaboration within communities while developing the testing centres. Decentralizing testing sites and moving them into communities was seen as an effective approach to increase accessibility, especially for disproportionately affected vulnerable people in society, including Indigenous people [36]. The above-mentioned decentralised testing solutions, including deployment of home test, driving thru testing, and local testing site, could be adopted to improve access to testing and reduce turnaround times. These are improvements that keep patients at the forefront by enabling greater access and faster results, minimizing excessive wait times and artificial crowds at test collection locations. Health system should plan with local community leaders and businesses to provide testing within the community. This may be best done through local pharmacies as studies found they can have a crucial role in community health and the response to COVID-19 [65, 66]. Increasing testing in pharmacies is a feasible, evidence-based approach for policymakers to consider. Increasing availability of testing sites within local communities was also a strategy to decrease the stigma associated with testing and is an appropriate option to increase accommodation for groups who lack trust in the health system and may feel anxious about visiting a centralized location.

### Strengths and Limitations

Strengths of this review include our comprehensive search strategy and inclusion of grey literature. As the COVID-19 pandemic continues to evolve, it was necessary to include the most up-to-date, current information. Several limitations of this review were identified. First, the majority of sources [39] were opinion pieces (i.e., commentaries, perspectives, media articles). Two sources were guidelines to prevent stigma and carrying out contact tracing, and twenty-one articles collected or analyzed primary or secondary data. These findings demonstrate a lack of experimental and observational designs to better understand the development of barriers to COVID-19 testing and to direct the impact of strategies. There is a need for both types of studies to determine the extent to which a barrier inhibits COVID-19 test seeking behaviour and the effectiveness of strategies to improve the uptake of COVID-19 testing or address testing hesitancy. Further, most publications were from the United States, which limits our understanding of testing barriers in other areas of the world. Finally, in the context of a rapid review, there are several limitations in the methods including lack of assessment of bias and evaluation of evidence. Caution should be used when interpreting the implications of the results.

## Conclusion

As testing capacity grows, policymakers and leaders have the chance to develop appropriate testing policies. Information from the review will help build the foundation for testing strategies that will help health systems respond to the COVID-19 pandemic most effectively. Although, the impact of effective strategies to address COVID-19 testing barriers or testing hesitancy is largely unknown, the information found within this review provides some evidentiary basis to suggest that multi-pronged approaches to addressing barriers are being both suggested and implemented.

## Supplementary Information

**Additional file 1.**   

## Data Availability

The data supporting the conclusion of this article is available upon reasonable request and the completion of a data transfer agreement.
