# Supplementary material for: Barriers to and strategies to address COVID-19 testing hesitancy: a rapid scoping review
Source: BMC Public Health. 2022 Apr 14;22:750. doi: 10.1186/s12889-022-13127-7 (PMC9008387; doi:10.1186/s12889-022-13127-7)
Supplement: Supplementary file 1 — Additional file 1. [file 12889_2022_13127_MOESM1_ESM.docx]

# Appendix A

**Search strategy details**

All database searches were executed on January 8, 2021.

**Ovid MEDLINE**

*COVID-19 filter: adapted from Ovid filter (*[*https://tools.ovid.com/coronavirus/Covid-19%20search%20notes.pdf*](https://tools.ovid.com/coronavirus/Covid-19%20search%20notes.pdf)*); SARS/MERS & HIV literature not expressly excluded*

| **#** | **Query** |
| --- | --- |
| 1 | exp Coronavirus/ |
| 2 | exp Coronavirus Infections/ |
| 3 | (coronavirus* or corona virus* or oc43 or nl63 or 229e or hku1 or hcov* or ncov* or covid* or sarscov* or sarscov* or sars-coronavirus* or severe acute respiratory syndrome coronavirus*).mp. |
| 4 | (or/1-3) and ((20191* or 202*).dp. or 20190101:20301231.(ep).) |
| 5 | ((pneumonia or covid* or coronavirus* or corona virus* or ncov* or 2019-ncov or sars*).mp. or exp pneumonia/) and Wuhan.mp. |
| 6 | (2019-ncov or ncov19 or ncov-19 or sars-cov2 or sars-cov-2 or sarscov2 or sarscov-2 or sarscoronavirus2 or sars-coronavirus-2 or coronavirus-19 or covid19 or covid-19 or covid 2019 or "2019-novel cov" or ((novel or new or nouveau) adj2 (cov or ncov or covid or coronavirus* or corona virus or pandemi*2)) or (coronavirus* and pneumonia)).mp. |
| 7 | covid-19.rx,px,ox. or severe acute respiratory syndrome coronavirus 2.os. |
| 8 | or/5-7 |
| 9 | 4 not (camel* or dromedar* or equine or coronary or coronal or covidence* or covidien or influenza virus or bovine or calves or tgev or feline or porcine or erinaceus or bcov or ped or pedv or pdcov or fipv or fcov or canine or ccov or zoonotic or avian influenza or h1n1 or h5n1 or h5n6 or ibv or murine corona*).mp. |
| 10 | 8 and (camel* or dromedar* or equine or coronary or coronal or covidence* or covidien or influenza virus or bovine or calves or tgev or feline or porcine or erinaceus or bcov or ped or pedv or pdcov or fipv or fcov or canine or ccov or zoonotic or avian influenza or h1n1 or h5n1 or h5n6 or ibv or murine corona*).mp. |
| 11 | or/8-10 |
| 12 | 11 and 20191201:20301231.(dt). |
| 13 | ((test* or screen* or pcr or rt-pcr or qt-pcr or lamp or contact tracing or contact investigation* or contact screening or contact epidemiology) adj5 (access* or attitud* or behavio?r* or aversion? or hesitanc* or objection? or oppos* or reluctan* or resist* or barrier* or deter or deters or deterred or difficult* or discourage* or hindrance* or hinder* or hurdle* or impediment* or obstacle* or (chang* adj2 mind?) or convinc* or galvani* or motivat* or persuad* or persuas* or ease? or easing or expedit* or facilitat* or help? or helping or promot* or equality or equit* or inequality or inequit* or fair or fairly or fairness or shame* or stigma*)).ti,ab,kw,kf. |
| 14 | ((test* or screen* or pcr or rt-pcr or qt-pcr or lamp or contact tracing or contact investigation* or contact screening or contact epidemiology) adj5 (population group* or ethnic group* or "sub group*" or ethnic population* or "sub population*" or ethnically diverse or poverty or impoverished or low income or low resource* or socioeconomic* or socio-economic* or ((rural* or remote* or isolated) adj2 (population* or area? or communit* or place?)) or "at risk" or minorit* or vulnerable or disparate* or disparit* or immigrant* or migrant* or foreign* or newcomer* or illegal alien* or expat* or ex-pat* or emigrant* or refugee* or indigenous or aboriginal* or first nations or native american* or maori? or african american* or black or bame or bme or "person* of colo?r" or "people of colo?r" or bipoc or latina? or latino? or latinx or latin american* or hispanic? or spanish speaking or lesbian* or gay* or bisexual* or homosexual* or queer* or same sex or same gender or sexual minorit* or trans or transgender* or transsexual* or lgbt* or glbt* or prison* or imprison* or incarcerate* or inmate* or convict* or jail* or correctional facilit* or detention facilit* or penitentiar*)).ti,ab,kw,kf. |
| 15 | 13 or 14 |
| 16 | 12 and 15 |

**Scopus**

MEDLINE results have been removed from Scopus results using *AND NOT INDEX(medline)*

| **#** | **Query** |
| --- | --- |
| 1 | ( ( TITLE-ABS-KEY ( coronavirus* OR "corona virus*" OR oc43 OR nl63 OR 229e OR hku1 OR hcov* OR ncov* OR covid* OR sarscov* OR sarscov* OR "sars-coronavirus*" OR "severe acute respiratory syndrome coronavirus*" ) ) AND NOT ( TITLE-ABS-KEY ( camel* OR dromedar* OR equine OR coronary OR coronal OR covidence* OR covidien OR "influenza virus" OR bovine OR calves OR tgev OR feline OR porcine OR erinaceus OR bcov OR ped OR pedv OR pdcov OR fipv OR fcov OR canine OR ccov OR zoonotic OR "avian influenza" OR h1n1 OR h5n1 OR h5n6 OR ibv OR "murine corona*" ) ) ) OR ( ( TITLE-ABS-KEY ( "2019-ncov" OR ncov19 OR "ncov-19" OR "sars-cov2" OR "sars-cov-2" OR sarscov2 OR "sarscov-2" OR sarscoronavirus2 OR "sars-coronavirus-2" OR "coronavirus-19" OR covid19 OR "covid-19" OR "covid 2019" OR "2019-novel cov" OR ( ( novel OR new OR nouveau ) W/2 ( cov OR ncov OR covid OR coronavirus* OR "corona virus" OR pandemic* ) ) OR ( coronavirus* AND pneumonia ) ) ) AND ( TITLE-ABS-KEY ( camel* OR dromedar* OR equine OR coronary OR coronal OR covidence* OR covidien OR "influenza virus" OR bovine OR calves OR tgev OR feline OR porcine OR erinaceus OR bcov OR ped OR pedv OR pdcov OR fipv OR fcov OR canine OR ccov OR zoonotic OR "avian influenza" OR h1n1 OR h5n1 OR h5n6 OR ibv OR "murine corona*" ) ) ) AND ( LIMIT-TO ( PUBYEAR , 2021 ) OR LIMIT-TO ( PUBYEAR , 2020 ) OR LIMIT-TO ( PUBYEAR , 2019 ) ) |
| 2 | TITLE-ABS-KEY ( ( test* OR screen* OR pcr OR "rt-pcr" OR "qt-pcr" OR lamp OR "contact tracing" OR "contact investigation*" OR "contact screening" OR "contact epidemiology" ) W/5 ( access* OR attitud* OR behavior* OR behaviour* OR aversion* OR hesitanc* OR objection* OR oppos* OR reluctan* OR resist* OR barrier* OR deter OR deters OR deterred OR difficult* OR discourage* OR hindrance* OR hinder* OR hurdle* OR impediment* OR obstacle* OR ( chang* W/2 mind* ) OR convinc* OR galvani* OR motivat* OR persuad* OR persuas* OR ease* OR easing OR expedit* OR facilitat* OR help* OR promot* OR equality OR equit* OR inequality OR inequit* OR fair OR fairly OR fairness OR shame* OR stigma* ) ) |
| 3 | TITLE-ABS-KEY ( ( test* OR screen* OR pcr OR "rt-pcr" OR "qt-pcr" OR lamp OR "contact tracing" OR "contact investigation*" OR "contact screening" OR "contact epidemiology" ) W/5 ( "population group*" OR "ethnic group*" OR "sub group*" OR "ethnic population*" OR "sub population*" OR "ethnically diverse" OR poverty OR impoverished OR "low income" OR "low resource*" OR socioeconomic* OR "socio-economic*" OR ( ( rural* OR remote* OR isolated ) W/2 ( population* OR area* OR communit* OR place* ) ) OR "at risk" OR minorit* OR vulnerable OR disparate* OR disparit* OR immigrant* OR migrant* OR foreign* OR newcomer* OR "illegal alien*" OR expat* OR ex-pat* OR emigrant* OR refugee* OR indigenous OR aboriginal* OR "first nations" OR "native american*" OR maori? OR "african american*" OR black OR bame OR bme OR "person* of color" OR "person* or colour" OR "people of color" OR "people of colour" OR bipoc OR latina* OR latino* OR latinx OR "latin american*" OR hispanic* OR "spanish speaking" OR lesbian* OR gay* OR bisexual* OR homosexual* OR queer* OR "same sex" OR "same gender" OR "sexual minorit*" OR trans OR transgender* OR transsexual* OR lgbt* OR glbt* OR prison* OR imprison* OR incarcerate* OR inmate* OR convict* OR jail* OR "correctional facilit*" OR "detention facilit*" OR penitentiar* ) ) |
| 4 | #2 OR #3 |
| 5 | (#1 AND #4) AND NOT INDEX(medline) |

**medRxiv and bioRxiv**

Advanced search; medRxiv and bioRxiv; Abstract or Title field; select "all"; 25 per page; Best Match; export top 25 results (or all available if less than 25 results) for each search as of January 8, 2021.

| 1 | "covid-19" testing access |
| --- | --- |
| 2 | "covid-19" testing attitudes |
| 3 | "covid-19" testing behavior |
| 4 | "covid-19" testing hesitancy |
| 5 | "covid-19" testing equity |
| 6 | "covid-19" testing minority |
| 7 | "covid-19" testing vulnerable |
| 8 | "covid-19" testing disparity |
| 9 | "sars-cov-2" testing access |
| 10 | "sars-cov-2" testing attitudes |
| 11 | "sars-cov-2" testing behavior |
| 12 | "sars-cov-2" testing hesitancy |
| 13 | "sars-cov-2" testing equity |
| 14 | "sars-cov-2" testing minority |
| 15 | "sars-cov-2" testing vulnerable |
| 16 | "sars-cov-2" testing disparity |

**Cochrane Database of Systematic Reviews (CDSR)**

| **#** | **Query** |
| --- | --- |
| 1 | ("2019-ncov" OR ncov19 OR "ncov-19" OR "sars-cov2" OR "sars-cov-2" OR sarscov2 OR "sarscov-2" OR sarscoronavirus2 OR "sars-coronavirus-2" OR "coronavirus-19" OR covid19 OR "covid-19" OR "covid 2019" OR "2019-novel cov" OR ((novel OR new OR nouveau) near/2 (cov OR ncov OR covid OR coronavirus* OR "corona virus" OR pandemic*)) OR (coronavirus* AND pneumonia)):ti,ab |
| 2 | ((test* OR screen* OR pcr OR "rt-pcr" OR "qt-pcr" OR lamp OR "contact tracing" OR "contact investigation*" OR "contact screening" OR "contact epidemiology") near/5 (access* OR attitud* OR behavior* OR behaviour* OR aversion* OR hesitanc* OR objection* OR oppos* OR reluctan* OR resist* OR barrier* OR deter OR deters OR deterred OR difficult* OR discourage* OR hindrance* OR hinder* OR hurdle* OR impediment* OR obstacle* OR (chang* near/2 mind*) OR convinc* OR galvani* OR motivat* OR persuad* OR persuas* OR ease* OR easing OR expedit* OR facilitat* OR help* OR promot* OR equality OR equit* OR inequality OR inequit* OR fair OR fairly OR fairness OR shame* OR stigma*)):ti,ab |
| 3 | ((test* OR screen* OR pcr OR "rt-pcr" OR "qt-pcr" OR lamp OR "contact tracing" OR "contact investigation*" OR "contact screening" OR "contact epidemiology") near/5 ("population group*" OR "ethnic group*" OR "sub group*" OR "ethnic population*" OR "sub population*" OR "ethnically diverse" OR poverty OR impoverished OR "low income" OR "low resource*" OR socioeconomic* OR "socio-economic*" OR ((rural* OR remote* OR isolated) near/2 (population* OR area* OR communit* OR place*)) OR "at risk" OR minorit* OR vulnerable OR disparate* OR disparit* OR immigrant* OR migrant* OR foreign* OR newcomer* OR "illegal alien*" OR expat* OR ex-pat* OR emigrant* OR refugee* OR indigenous OR aboriginal* OR "first nations" OR "native american*" OR maori* OR "african american*" OR black OR bame OR bme OR "person* of color" OR "person* or colour" OR "people of color" OR "people of colour" OR bipoc OR latina* OR latino* OR latinx OR "latin american*" OR hispanic* OR "spanish speaking" OR lesbian* OR gay* OR bisexual* OR homosexual* OR queer* OR "same sex" OR "same gender" OR "sexual minorit*" OR trans OR transgender* OR transsexual* OR lgbt* OR glbt* OR prison* OR imprison* OR incarcerate* OR inmate* OR convict* OR jail* OR "correctional facilit*" OR "detention facilit*" OR penitentiar*)):ti,ab |
| 4 | #2 or #3 |
| 5 | #1 and #4 |

**Google**

Screening protocol: Go 2 pages (20 results) beyond the last result clicked

| " "covid-19" \| coronavirus \| "sars-cov-2" " " test \| "contact tracing" " " attitude \| behavior \| aversion \| hesitancy \| objection \| oppose \| reluctance \| resistance " |
| --- |
| " "covid-19" \| coronavirus \| "sars-cov-2" " " test \| "contact tracing" " " barrier \| deterred \| difficulty \| discouraged \| hindered \| hurdle \| impediment \| obstacle " |
| " "covid-19" \| coronavirus \| "sars-cov-2" " " test \| "contact tracing" " " change minds \| convince \| galvanize \| motivate \| persuade " |
| " "covid-19" \| coronavirus \| "sars-cov-2" " " test \| "contact tracing" " " ease \| expedite \| facilitate \| help \| promote " |
| " "covid-19" \| coronavirus \| "sars-cov-2" " " test \| "contact tracing" " " equality \| inequality \| equity \| inequity \| fairness \| shame \| stigma " |
| " "covid-19" \| coronavirus \| "sars-cov-2" " " test \| "contact tracing" " " "population group" \| "ethnic group" \| "sub group" \| "ethnic population" \| "sub population" \| "ethnically diverse" " |
| " "covid-19" \| coronavirus \| "sars-cov-2" " " test \| "contact tracing" " " poverty \| impoverished \| "low income" \| "low resource" \| socioeconomic \| rural \| remote " |
| " "covid-19" \| coronavirus \| "sars-cov-2" " " test \| "contact tracing" " " "at risk" \| minority \| vulnerable \| disparate \| disparity \| immigrant \| migrant \| foreign \| newcomer \| refugee " |
| " "covid-19" \| coronavirus \| "sars-cov-2" " " test \| "contact tracing" " " indigenous \| aboriginal \| "first nations" \| "native american" \| maori " |
| " "covid-19" \| coronavirus \| "sars-cov-2" " " test \| "contact tracing" " " "african american" \| black american \| "african canadian" \| black canadian \| black people \| bame \| bme \| "people of color" \| "people of colour" \| bipoc " |
| " "covid-19" \| coronavirus \| "sars-cov-2" " " test \| "contact tracing" " " latina \| latino \| latinx \| "latin american" \| hispanic \| "spanish speaking" " |
| " "covid-19" \| coronavirus \| "sars-cov-2" " " test \| "contact tracing" " " lesbian \| gay \| bisexual \| homosexual \| queer \| "same sex" \| "same gender" \| "sexual minority" \| trans \| transgender \| transsexual \| lgbt \| glbt \| lgbtq \| lgbtq+ \| lgbtq2+ " |
| " "covid-19" \| coronavirus \| "sars-cov-2" " " test \| "contact tracing" " " prison \| imprisoned \| incarcerated \| inmate \| convict \| jail \| "correctional facility" \| "detention facility" \| penitentiary " |

**Other Grey Literature Sources**

The following list of websites were searched for grey literature: OECD, WHO, CDC, ECDC, CADTH, National public health websites (e.g., Australia, UK, New Zealand, United States), Coronavirus resources (e.g., Johns Hopkins, COVID-END, CAN-COVID, CORD19).

#

# Appendix B: Inclusion/exclusion criteria

| **Include/Exclude** | **Criteria** |
| --- | --- |
| Include | COVID-19 |
| Include | Population: All demographics and population subgroups eligible |
| Include | English language |
| Include | Setting: any setting related to COVID-19 testing OR any learnings from previous infectious disease-related epidemics or pandemics applied to the COVID-19 response. |
| Include | Study design:  Published and pre-print pieces for academic journals (social science, science, and medicine); Research articles (multiple designs not just randomized control trails should be within scope); Letters; Commentary/perspectives/editorials; Grey literature (e.g. government, non-profits, etc.) |
| Include | Interventions: barriers to testing and strategies to address barriers to testing. Article should focus on individual behaviour/attitudes toward COVID-19 testing in any setting or population (ex. testing hesitancy, access to tests, access to information, economic impact of testing, the impact of repeat testing, and inequity) OR Article should focus on strategies to address individual behaviours toward barriers to testing |
| Exclude | non-COVID-19 |
| Exclude | Non-English papers |
| Exclude | Purely technical papers; sources that only focus on technical aspect of testing (efficacy, protocols, procedures, etc.)  Sources that only focus on testing outcomes  Sources on general testing strategies that do not address barriers to testing |

# Appendix C: PRISM Diagram of included and excluded studies
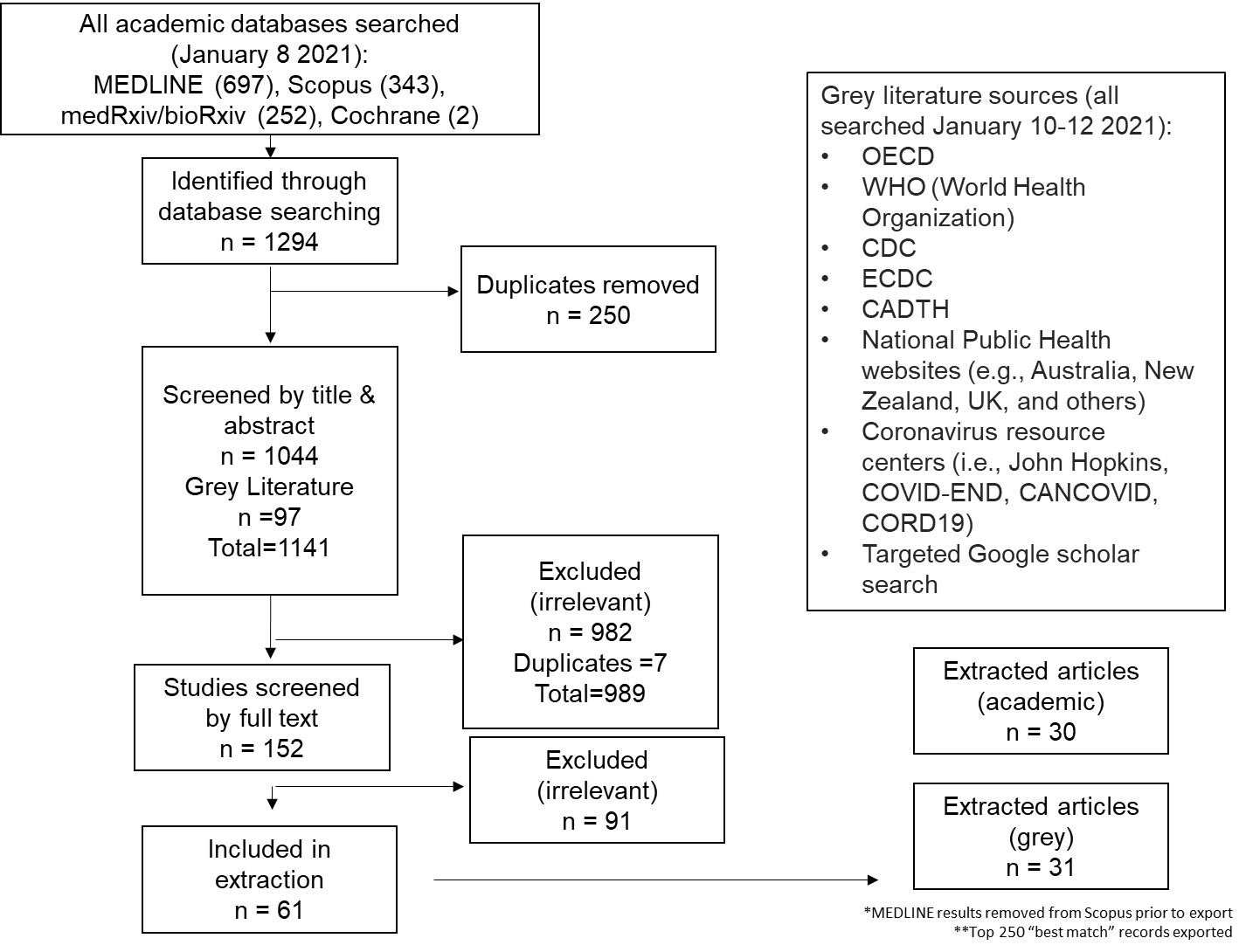


**Appendix D: Annex**

| **Author-year and reference** | **Journal/ source** | **Country** | **Source** | **Purpose** | **Type/**  **design** | **Barriers identified** | **Strategies suggested or implemented** |
| --- | --- | --- | --- | --- | --- | --- | --- |
| Adebisi 2020 | American Journal of Tropical Medicine and Hygiene | Africa | Academic publication | Perspective on sex workers in Africa access to testing during COVID-19 | Perspective | Social stigma, gender-based violence, and discriminatory policies inhibit access to care and treatment.  This also hinders contract tracing. Reducing stigma will provide opportunities. to seek care and preventative measures.  Sex workers are mobile and do not receive targeted information.  Sex workers do not have access to many social services, such as health insurance. | “examine the disadvantages people face, to empower those who are left behind, and to enact inclusive far-sighted and progressive sustainable development goals."  Involve communities in social protection schemes, health services. |
| Adeniji 2020 | African Journal of Primary Health Care and Family Medicine | South Africa | Academic publication | A review of the literature to assess the available means of wide-scale testing for COVID-19 in order to positively affect the testing rate. | Narrative review | The author describes the rate of testing for COVID-19 in South Africa and overviews alternative testing methods, mostly self-administered, as a means of increasing testing availability. | The author outlines some recommendations that may be implemented in South Africa to increase the testing rate in the country, including distributing self-administered COVID-19 tests, providing education to individuals on how the tests can be administered, and studying the effects of such interventions. |
| Atchison 2020 | Clinical Infectious Diseases | UK | Academic publication | To examine self-administered SARS-CoV-2 antibody testing in the home setting to determine its usability and acceptability.  Lateral flow immunoassays were the tests used. | Cross sectional design  (n= 315 in pilot; 8754 of 10600 who received LFIA1 kits; 2957 of 3800 who received LFIA2 kits) | Testing acceptability. | None. |
| Associated Press 2020 | Media | United States | Media | Reports on test disparities in Phoenix, Arizona, specifically in Black and Latino neighbourhoods. Specifically, the article details barriers in opportunity for receiving a test surrounding racial disparities, and structural barriers. | Text and opinion | The barriers to testing reported surround racial disparities and inequities in the healthcare system. Specifically, there was an anecdotal report of hundreds of people lining up to receive a test and having to wait hours.  Anecdotes from Pheonix describe nowhere to go and get tested, long wait times, and testing sites being set up in wealthy neighbourhoods. A free testing drive-thru event was scheduled and was not well received. This was because there was such high demand, the wait time was 13 hours, highlighting that people are willing to get tested, but the infrastructure is not there to support them. | Free testing and more testing for all. |
| Babych  2020 | Media | Canada | Media | Article describes a research project in Alberta that is piloting the delivery of medical supplies, including COVID-19 test kits, to remote, First Nations communities using drones. | Media article, research design unknown | Living in a remote community. | The pilot is being done to test the means of delivering medical supplies to remote communities during the COVID-19 pandemic.  Currently many of these communities across Canada have limited access to testing centres and related medical supplies that can support rapid testing. |
| Bartlett 2020 | Media | United States | Media | Media article focusing on the cost of a COVID-19 test as a barrier for testing and travel.  Article mostly describes the experience of a woman trying to get tested in order to travel, but vulnerable populations are briefly mentioned as not receiving adequate testing. | Media article | The majority of the article is not necessarily focused on vulnerable populations,  however there is short mention of communities of color and low-income communities and that they should be given priority. The barriers to testing presented in the article are mainly to do with a lack of insurance to cover the cost of the test, in addition to wait time. | Federal funding to cover the costs of testing can reduce the cost of testing burden on insurers and control premiums for consumers. |
| Bonner et al 2020 | medRxiv | Australia | Preprint | The aim is to address a major gap in understanding how to improve COVID-19 testing behaviour, by: 1) reporting the prevalence of specific test barriers via survey; 2) identify additional test barriers through open responses; and 3) linking barriers to an overarching framework of behaviour change. | Longitudinal survey  (n=4326 participants; testing barriers of interest were included in the Wave 3 study – n=1369 participants) | Testing is painful (n=153; 11.2% of respondents)  I don’t know when to get tested (n=98, 7.1% of respondents)  Worried about getting infected at a testing clinic (n=81; 5.9% of respondents)  I’ll forget to get tested (n=33; 2.4% of respondents)  Other: (organized in behaviour change theory)  Worried about what others think (stigma) (n=33, 2.4%)  Too difficult, too expensive (n=32, 2.3%)  Testing doesn't work (n=17, 1.2%)  No one else is getting tested (n=11, 0.8%) | None specifically provided |
| Capps & Gelatt  2020 | Think Tank | United States | White paper | The authors described barriers to COVID-19 testing for uninsured immigrants in different states. They undertook an analysis of the American Community Survey. | Policy analysis | Insurance inadequacy to cover the costs of testing. The authors mention that some steps have been taken to cover the cost of testing but that other options for covering the cost of tests exists. While this barrier is not described in depth and there is more investigation presented on understanding unemployment and un-insurance. | Federal government provide incentive for states to individually provide Medicare.  Authors suggest that a program, similar to Medicaid, be created specifically for lawful protected resident adults who are excluded from Medicaid.  Opportunity for public/private partnerships in order to get funding for community health centers to provide coverage for those in need. |
| Clipman  2020 | medRxiv | United States | Preprint | A survey of residents of Maryland, Florida, and Illinois to assess access to and barriers for testing. | Cross-sectional survey  (n=3058 participants completed survey) | n=146 respondents wanted/needed a test, but did not get tested, main reasons were: 1) not knowing where to go (36%); 2) distance/waiting time (33%); and 3) 21% reported fear of being tested.  A further n=177 respondents reported symptoms, exposure or both but did not want a test. Main reasons for this were: 1) belief that symptoms were due to other causes (42%); 2) no symptoms (18%); 3) not wanting to know one’s status (18%); and 4) logistic issues, such as not knowing where to go or lack of transportation (15%). | Clear communication strategies and messaging.  Access to rapid, POC, COVID-19 testing for results expediency. |
| Cordes & Castro 2020 | Spatial and Spatio-temporal epidemiology | USA | Academic publication | Spatial analysis of the rates of testing for COVID-19 and percent positivity through a spatial analysis of zip code data for the City of New York.  The authors were interested in determining whether certain population characteristics were associated with low levels of testing and high positivity rates among areas of New York City. | Spatial analysis  (n=177 zip codes) | The authors found that there were geographic clusters of areas that had lower rates of testing and positivity, as well as those areas with high rates of testing and positivity. The former areas tended to have higher income and educational attainment, as well as a larger White population. The latter areas tended to have more individuals with no health insurance and a larger Black population. | None provided |
| COVID-19 Unified  Command  2020 | Government Website | United States | Grey literature | Guidance developed for testing and contact tracing using a health equity approach | Guidance document | Many barriers were identified and described including: 1) low health literacy; 2) testing site location or hours of operation; 3) physical, cognitive, developmental or functional limitations; 4) communication and transportation barriers also play a role in accessing COVID-19 testing services; 5) socieconomic barriers affect post-COVID-19 testing supports, ability to self-isolate; access to paid leave and time spent away from work (if testing positive), paying for childcare to attend a testing event.  Some people may be underhoused or fear immigration retribution and may not have permanent contact information or disclose this, which impacts on contact tracing efforts.   There is a fear of the cost of the test, the need for medical insurance to get a test, and fear of medical costs if you become sick with COVID-19.  Cultural and social barriers are also outlined. | Offer bilingual, culturally-tailored testing and contact tracing services in communities most in need. Focus on communities with elevated risk - select communities for testing using a data-driven approach and tailor testing events (outreach, approach and accommodations).  Consider processes to include individuals who lack permanent contact information or have unstable housing.  Select testing format to be inclusive of barriers such as transportation.  Suggest using a mixed approach to meet as many needs as possible.  Testing formats include: drive-thru, walk-up site, mobile screening (service meets participants at a predetermined location but moves to another location), door-to-door - meeting participants in their homes.   Consider testing hours and locations.  Recognizable location that is familiar to the community and large enough to accommodate the format, close to public transportation.  Ensure testing locations are accommodating for all abilities. Work in collaboration with a range of community groups that advocate and serve people from different cultures, races, ethnicities, across varying languages and abilities.  Hours should be inclusive of those with non-traditional work schedules (outside of just 9-5pm).  Seeking input from communities to ensure that tests are equitable. |
| Staff DD  2020 | Media | Ireland | Media | Media article discusses COVID-19 testing stigma in Ireland from the perspective of GPs. | Text and opinion | People are not seeking testing because they are afraid of being judged. | None provided. |
| Dodds & Fakoya  2020 | BMJ | UK | Academic publication | Editorial to advocate for the inclusion of ethnic minority groups in COVID-19 testing programs. | Editorial | Based on their previous research on other diseases, the authors outline the potential for mistrust between some ethnic minorities and the government when it comes to testing. | The needs of ethnic minority groups are required to be met to ensure the success and uptake of testing programs. This can be done through building trust by increasing access to testing options and providing equitable health care where needed.  They advocate for including community representatives in the planning of COVID-19 testing initiatives to build trust and strengthen the uptake of testing. |
| Doyle 2020 | CMAJ | Canada | Academic publication |  | Text and opinion | Various barriers for migrant workers in Canada are identified: 1) access to healthcare; 2) inability to work if testing positive for COVID-19. | Permanent and universal coverage for all medically necessary services regardless of immigration status. |
| Dryden-Peterson  2020 | medRxiv | United States | Preprint | With improved testing infrastructure in Massachusetts, the study team sought to examine how SARS-CoV-2 testing aligned with the intensity of the epidemic. | Prevalence study  4,262,000 tests were reported in the time period under investigation. | Community socioeconomic vulnerability. | Strategies are not provided, other than to recognize that addressing structural inequities will help support alignment between epidemic intensity and resourcing of testing for future COVID-19 pandemic. |
| Earnshaw  2020 | Stigma and Health | United States | Academic publication | This study explored whether anticipated stigma and stereotypes are associated with the likelihood that one will get COVID-19 testing. | Cross-sectional study  (n=845 participants that met inclusion criteria; 77.0% identified as White; 10.2% identified as Black, 3.6% identified as Asian; 3.0% identified as Latino(a) and 6.3% identified as "other" | The perception that one will experience discrimination based on a stigmatized status.  Research from other infectious diseases suggest that people will avoid engaging in testing or treatment because of stigma associated with this.  In this study, those persons with greater anticipated stigma and agreement with COVID-19 stereotypes were less likely to get a COVID-19 test.  Study suggests that anticipated stigma may play a role in whether someone will get a COVID-19 test. | Authors suggest strategies to address stigma include mass media and educational interventions. Authors also suggest the expansion of non-discriminatory policies to include COVID-19. |
| Egelko  2020 | American Journal of Public Health | United States | Academic publication | Commentary describes a number of barriers to mandatory testing in racialized communities and presents considerations for the ethical implementation and uptake of testing in this population. | Text and opinion | Multiple barriers described including: repercussions, research aversion and mistrust of science as contributing to testing hesitancy in racialized communities in the United States of America.  In addition, financial barriers as testing can lead to mandatory quarantine of individuals with no sick leave or benefits. Immigration status is also highlighted as a barrier in the US as use of public resources can be counted against individuals throughout the immigration process. Stigma was also described a barrier to testing in these already marginalized communities. Other barriers include financial stressors; immigration status; and stigma/stereotype threat. | Community trust needs to be fostered with the aim of making testing an attractive option for all individuals. Specifically, the authors suggest testing as a surveillance method which allows for anonymity instead of as a case-finding method which can be infringing. |
| Evans  2020 | New England Journal of Medicine | United States | Academic publication | Perspective article on how COVID-19 is disproportionately impacting  African Americans, Latinx Americans, and Native Americans. | Perspective | Mistrust in the health care system: Health care providers, health care organizations, and academic medical centers should consider how their attitudes, actions, management, and ignorance of the realities that shape the lives of minority populations contribute to health disparities. | Several strategies are suggested to support populations with testing uptake including: free tests, contact tracing, shorter reporting time, and free temporary housing for folks who need to isolate. |
| Feldman 2020 | Media | United States | Media | This news media article to describe a free testing initiative in Philadelphia offered by the Black Doctors COVID-19 Consortium. The article goes in-depth into the barriers faced by Black people with respect to COVID-19. | Text and opinion | The following are a list of reasons (why COVID-19 prevalence is higher in Black communities) briefly mentioned in the news article (verbatim): a.) Black people are less likely to have primary care physicians b.) Black people are more likely to rely on public transportation c.) Black Philadelphians are more likely to work jobs that can't be performed at home, putting them at a greater risk of exposure d.) in jail's, sanitation, and transportation departments, workers are predominantly Black e.) the increased severity of illness among Black people may be due to underlying health conditions. | Free testing was set up in order to help curb the burden of COVID-19 on Black people in Philadelphia. It is briefly mentioned that lack of insurance may inhibit an opportunity for a test as the cost associated with a test may be too much. |
| Fernando 2020 | Media | United States | Media | To describe the experiences of Americans in seeking COVID-19 testing. | Text and opinion | This article outlined several barriers to testing that Americans face, particularly: test shortages, cost of testing, cost of missing work and accessibility of testing sites. | They highlight the need for more rapid, affordable and accessible COVID-19 tests. |
| Fleming  2020 | Nature | Global | Academic publication | Article identifies ways that scientists and individuals can tackle misinformation about COVID-19. Some expert opinion is provided. | Text and opinion | Misinformation on the pandemic. | Scientists are in a place to tackle misinformation by countering it with facts. They can call out fake conspiracy theories and stories.  Various strategies for individuals to use when reading to spot misinformation: Source suspicion, bad language, emotional contagion, eureka news, false accounting, oversharing, money conflict, fact check. |
| Fusco  2020 | AIDS | Italy | Academic publication | Commentary focuses on the potential for underdiagnosis of COVID-19 among persons living with HIV (PLWH).  Reports on the number of PLWH who completed COVID-19 nasopharyngeal swabs over the period of the time of the start of the epidemic to April 22, 2020. | Commentary  (n=12,653 patients; n=16,382 NP swabs – of these only n=12 PLWH completed an NP swab) | Fear of COVID-19 transmission: Regularly, 2392 PLWH receive care from the hospital (routine blood work, therapy withdrawal) and only 0.5% of this population of patients referred for general COVID-19 testing.  The authors speculate that since PLWH represent a high-risk group, there is a hesitancy to use community health services (for fear of COVID-19 transmission).  96% voluntarily withdrew from usual services but unsure what their motivations are.   Persistent stigma within this population may also contribute. | Integrating COVID-19 services with usual HIV services.  Suggested by authors as an approach that is familiar to PLWH.  Ongoing screening of PLWH for active/recent symptoms of COVID-19 with testing as appropriate. |
| Galaviz 2020 | Health Equity | United States | Academic publication | This commentary describes how implementation science can support the development of strategies aimed at mitigating health disparities experienced by African American, Hispanic and Native American populations during the COVID-19 pandemic and into recovery.  These populations are overrepresented among both COVID-19 cases and COVID-19 deaths. | Perspective | Barriers described include: 1) language and health literacy; and 2) access to a testing site – gives the example from the state of Texas, where testing facilities are located predominantly in communities with "whiter" populations. | Designing equitable interventions.  These interventions should consider factors such as culture, history, values and needs of minority communities.  Suggests that testing should be deployed in places where minority communities both live and work. |
| Gillam 2020 | Journal of Public Health | UK | Academic publication | Describes the pilot of a mass testing initiative at a university setting and assessed its acceptability, costs, and ability of being scaled up to serve the testing needs of students and staff on a wider basis.  The authors evaluated a testing strategy in a setting with high-risk for infection and transmission (university) due to the possibility of asymptomatic cases. | Cross sectional study  (n=1053 registered individuals of whom 798 provided one or more swabs while 687 provided four swabs; n=458 participated in the post-study survey)  Copan Eswabs were used for all participants. | Testing acceptability.  Overall acceptability was high 4.5/5 (5 being most favourable). 97% reported they would participate in repeat testing.  71% agreed or strongly agreed that taking the swab was easy to do. |  |
| Hengel 2020 | The Lancet Infectious Diseases | Australia | Academic publication | In this descriptive paper, the authors described the enablers and challenges of setting up point-of-care testing in remote communities to increase access to testing among Indigenous peoples in Australia. | Viewpoint  (n=86 communities) | Availability and timeliness of COVID-19 testing in remote Australian communities. In light of the COVID-19 pandemic, the original guidelines for remote communities specified that individuals suspected of being infected were to be airlifted while awaiting for their results. To increase the availability of testing, the authors describe a model of point-of-care testing that was rolled out and which was based on an existing framework of testing for sexually transmitted infections. | Decentralized POC PCR testing was implemented. |
| Huerto 2020 | Health Affairs | United States | Grey literature online source | To describe how targeted strategies may help improve testing uptake. | Commentary |  | Targeted testing strategies include:  -testing resources toward people. with underlying medical conditions to make it safer to get tested.  -targeting people living in areas where there is physical distancing and other safe conditions.  -targeting testing towards essential workers.  Targeted messaging and clear, transparent criteria for testing (simple explicit instructions). |
| Ibarra 2020 | Media | United States | Media | Article describes the role that contact tracers have in offering COVID-19 tests to those who want one. Through discussing testing with experts in the field, this article reports on solutions to contain COVID-19 cases in California.  Article is focused on the Black and Latino communities of Oakland, California. | Text and opinion | Describes a lack of trust in the system, highlighting racial disparities, accompanied by structural barriers inhibiting Black and Latino populations from accessing testing. | Blanket testing – specifically among essential workers and/or seniors. |
| Jacobson 2020 | International Journal for Equity in Health | Global | Academic publication | This commentary specifically explores Covid-19 responses related to: 1) testing and surveillance; 2) contact and location tracing; 3) public mask use; and 4) social distancing, as well as unintended consequences of Covid-19 policies to ensure not only an equitable pandemic response but also a more equitable society in the post-pandemic era. | Commentary |  | The authors highlighted implementation strategies to be applied to testing, including: collecting disaggregated data to inform the need for adopting testing strategies, involving community leaders and community-based organizations to develop and coordinate testing strategies, and fostering engagement and public trust. |
| Jegede 2020 | Pan African Medical Journal | Nigeria | Academic publication | Commentary describes the ethical issues in providing adequate pandemic preparedness in locations that are resource limited, such as Nigeria. | Commentary | Stigma from COVID-19 positive test  Testing inequity – focused on testing “elites”  Lack of incentive for persons who test positive to identify contacts. | The authors describe the need for education and public awareness regarding prevention, the availability of free testing, and ensuring that isolation facilities, adequate amenities, and access to sanitization and disinfectants exists. |
| Kelly 2020 | Media | Canada | Media | The current piece is a news media article published on December 10, 2020. The aim of the piece is to describe the experiences of an Indigenous woman in Vancouver trying to seek testing for COVID-19. | Text and opinion | Clinic staff denied a test to an Indigenous woman meeting testing criteria (they suspected influenza). In addition, the article mentions that the family decided to travel to Tofino Hospital via water taxi in which case geographical barriers may also exist. | None |
| Kernberg 2020 | Obstetrics and Gynecology | United States | Academic publication | The goal was to determine COVID test uptake and reasoning for declining tests within the labour and delivery unit of a major hospital. | Cross sectional  Study  (n=289 eligible participants; n=270 accepted asymptomatic testing) | Testing discomfort. The authors identified discomfort as the main barrier to testing for of participants who declined a COVID-19 test across all phases of the study [63% (n=10/16) in phase 2; 75% (n=3/4) in phase 3; 66% (n=2/3) in phase 4)  Belief that the participant does not have COVID-19. This was identified by 13% (n=2/16) of participants who declined.  3/16 (19%) of participants declined the study for unknown reasons. | No strategies identified. |
| Khalidi 2020 | NGO website | Malaysia | Short report | Overview of why illegal citizens do not seek care during covid-19, including testing. | Research report | Illegal migrants fear of being charged if they are found.  Language is also a barrier, as they may not understand medical instructions or treatment options. They may also not understand how or where to seek care.  Fear of losing work if sick, as they rely on daily wages | Government initiatives that address misconceptions about what will happen when seeking COVID-19 testing.  Increasing engagement with stakeholders    including employers, embassies and NGOs to spread the message on what foreign workers must do to protect themselves. Outreach through social media may help. |
| Kissam  2020 | Statistical Journal of the IAOS | United States | Academic publication | Article addresses the challenges faced by local public health agencies in California to support their response to the COVID pandemic. | Case report |  | Argues that COVID-19 testing in local areas should focus less on large-scale use of COVID testing and instead shift towards using testing as a component of a more comprehensive public health strategy that includes testing, contact tracing for those who test positive, and additional supports for those required to quarantine/self-isolate. |
| Konkol  2020 | Media | United States | Media | Focuses on health system inequities for Black communities during the pandemic. Specifically, the article describes a situation in Chicago in which Governor Pritzker committed to providing tests and resources to Black communities, but fell short. | Media | Gap in testing of Black communities in the Chicago area (unclear if this is access to tests or other barriers). | Testing site focused on serving Black populations. |
| Lan  2020 | Frontiers in Public Health | China | Academic publication | Study was conducted to explore knowledge/attitudes/use of COVID-19 testing kits among those living in China (general public). | Cross sectional study  (n=1167 participants) | Across participants, there was good/decent knowledge and good attitudes towards testing. However, there was overall limited knowledge of tests beyond the traditional PCR test, including antigen and antibody tests. |  |
| Levitt  2020 | Media | United States | Media | Focus was on innovative ideas to persuade Americans to get tested for COVID-19. | Text and opinion | Cost of testing and testing positive for COVID-19 were identified as barriers. | Making testing free and readily accessible.  Suggests using incentives such as weekly prizes and providing a COVID dividend to people who test positive. |
| Li  2020 | Journal of Medical Internet Research | United States | Academic publication | Study aims to identify potential associations between demographic variables, internet use and COVID risk awareness and engaging in preventative behaviours (e.g. mask wearing, hand washing) and COVID testing behaviours. | Cross sectional study  (n=979 participants) | Authors identified that females were less likely to get a COVID test compared to male participants. The authors also identified that study participants without a partner or who were not married were less likely to get a COVID-19 test.  Li et al., (2020) did not identify a significant association between viewing COVID health information online and improvements in COVID testing behaviours. (OR= 10.3) |  |
| Loyola University  2020 | University website | United States | Media | To review COVID Equity Response Collaborative's activities to address equity. | Media | Barriers identified included: 1) cost of testing; 2) understanding testing; and 3) knowing how to access testing. | Building a multi-disciplinary network of academic, community, public, and other partners to help understand and address those most impacted by structural inequity, with a focus on testing and tracing. |
| Mahase  2020 | BMJ | UK | Media | News story of UK's Operation Moonshot, which proposed using general practices and pharmacies to make COVID-19 testing more available to the public. | Text and opinion | Testing is not available to the public. | Saliva tests would be a big step forward for patients. |
| Martin  2020 | Media | Canada | Media | News article about stigma of COVID-19 testing | Text and opinion | Stigma of COVID-19 testing that testing is painful | Communication and outreach to decrease stigma and educate on the test: Health professional who received the PCR test had media availability to speak to their firsthand testing experiences. |
| Maxmen  2020 | Nature | United States | Media | Interview with Dr Noha Abolelata who works with the non-profit organization Roots Community Health Centre in Oakland, California on COVID-19 disparities in Black American communities.  The interview summarizes the strategies employed in two regions of California experiencing disparities from COVID-19 and other health issues. | Text and opinion | Access to COVID-19 testing: People may not have access to a car.  Also residents of the communities are not comfortable accessing health services outside of their communities. (1) Systemic and historical racism:  Describes the experimentation of Black people (Tuskegee, syphilis study).  Researchers that have historically come to the community for data collection but there is no further engagement.  The idea of "insiders" taking information out.  Identification cards and email address: Some people do not have access to these things for usual testing processes.  Some do, but are mistrustful of the system so they don't want to provide them.  Communications: There is conflicting COVID-19 information leading to mistrust within the community.  There is the potential for information overload with a lack of clarity. | A multi-pronged approach is being used to augment access to testing.  This includes testing at physical clinics, youth shelters and by deploying a street medicine team (using a mobile RV) - which is also useful for contact tracing for those individuals who are underhoused.  Walk up testing sites have been set-up in both Oakland and San Jose.  Test samples are being sent to six different testing labs to ensure results are received in a timely manner.  Drive thru testing will not work for people who don't have access to a vehicle. Similar to the approach used for access to testing, having multiple options for testing facilities in the community acts like an "anchor" - residents are familiar with the health providers/clinics and "see people who look like them". Support network developed for those who test positive.  This includes ensuring shelter so people can self-isolate and quarantine, coordinating food delivery (food boxes) and providing cleaning supplies.  Ensuring there is a support network who can take groceries to them when required. |
| Minnesota Department of Health  2020 | Government website | United States | Grey literature | Identifying barriers and strategies for disabled people to access testing. | Guidance | Physical disability including limited mobility; blindness; low vision; difficulty hearing, communicating, or understanding information; and in some cases sensory challenges. | Ease physical access, reduce physical barriers.  More signage for those who may have visibility issues.  Reduce sensitivity issues, such as lights, noises, smells, etc.  Provide clear, easy to understand information. Allow them to get tested at their own pace.  Suggests that at-home testing may be an option for those living with disabilities and sensitivities.  May need additional support staff. Transportation to and from testing sites may be needed for people with disabilities and unique health needs. |
| Mitchell et al 2020 | Journal of the American College of Surgeons | United States | Academic publication | To describe the key contributors to the effective response to the COVID-19 pandemic in Western Washington. | Consensus statement from expert panel | The expert panel included accessibility and availability of testing as one of their success factors during the initial timeline of the pandemic. | In terms of lessons for the future, the authors emphasized the need for collaboration between laboratories and academia to ensure surveillance and population wide testing. |
| Mukattash  2020 | International Journal of Clinical Practice | Jordan | Academic publication | The present study aims to exploring community pharmacists’ willingness and readiness to test for COVID-19 in Jordan. | Qualitative interviews with 20 community pharmacists | Jordanian pharmacists are willing to test patients for COVID-19 in community pharmacies and in at-home testing.  Pharmacies are an accessible testing site for the public.  However, participants described they were not prepared/skilled administer tests and needed extra training and better safety precautions.  Physical space in pharmacies in Jordan is also a barrier. | Certification guidelines and programmes and clear legislations to govern such services in community pharmacies. |
| Murphy  2020 | Media | United States | Media | To describe a testing program for SEPTA employees. | Media | Access to testing for essential workers. | Offering free tests to targeted populations. |
| News  2020 | Media | United States | Media | Articles provide a description of the newly launched Black Doctors (COVID-19 Consortium whose initiative is to offer free mobile COVID-19 testing to at-risk and vulnerable communities. | Media | A lack of access to free testing is contributing to high rates of COVID-19 among Black communities. | Describes an initiative to offer free mobile testing to underserved/vulnerable communities. People who want a COVID-19 test, can sign up for an appointment online, as the group is testing anyone with symptoms or who has recently been in contact with a known positive. The consortium also has a GoFundMe page dedicated to generating money to continue providing the free testing, advocacy, and also a COVID-19 education component for Black persons. |
| OHSU  2020 | University website | United States | Media | Article outlines the novel strategies being used at Oregon Health & Science University to provide low-barrier testing, including reserving the first two hours for OHSU staff, their family members and first responders. In addition, if a patient does not have insurance, they can still have the test. | Media article | Not specific but talks about “low-barrier testing” strategy. | During high-volume periods, the first two hours of testing will be dedicated to first responders (police, firefighters, and emergency medical technicians), Oregon Health & Science University (OHSU) health patients and household members of OHSU health employees.  Bill insurance for those who have it and free tests to uninsured patients.  Expanded hours of testing and the introduction of pop-up mobile testing sites. |
| Page  2020 | New England Journal of Medicine | United States | Academic publication | Focuses on COVID-19 challenges experienced among the Latinx community. | Perspective | Demand for testing couldn't be met with available resources.  The public questioning the benefit of testing as there's no treatment.  Positive results could lead to job loss, isolation, stigmatization, and eviction. | Cash benefits should be extended to anyone who needs them, regardless of immigration status. |
| Quest Diagnostics  2020 | Media | United States | Media | Reports on the results of the Quest Diagnostics Health Trends Survey on Testing Hesitancy | Survey  (n=2050 adults including n=337 identifying as Hispanic or Latinx; n=265 identifying as Black; and n=1278 identifying as White) | Multiple barriers were identified, including: 1) Fear of getting infected; 2) Not believing they may have the virus; 3) Fear of quarantine; 4) Cost.  More Hispanic/Latinx adults (83%) than Whites(non-Hispanic (72%) and Blacks(72%) avoided or delayed a COVID-19 test when they believed they needed one. | The health system needs to design better communication strategies to promote testing. |
| Rader  2020 | medRxiv | United States | Preprint | To assess the travel time to testing sites to determine their geographic accessibility in the United States, specifically focusing on travel time under and over 20 minutes. | Spatial analysis  (n=6236 testing sites in 3108 counties) | Increased travel distance has the potential to decrease the geographic accessibility to testing. |  |
| Siegler  2020 | Open Forum Infectious Diseases | United States | Academic publication | To assess patient willingness to use various SARS-CoV-2 testing modalities for clinical care: home-based specimen collection, drive-through testing, and clinic-based testing. | Survey  (n=4593 eligible participants; only n=1260 respondents) | Public preference for Covid-19 tests based on ease of use.  Home testing was the most preferred type of test (92%), followed by drive-thru (71%), then lab or clinic-based tests. Preference differences were not affected by onset of symptoms, age, race or location. |  |
| Singh  2020 | Medical Decision Making | United States | Academic publication | To examine whether providing COVID-19 testing at United States Postal Service locations would increase access by reducing the driving distances.  The authors undertook a modeling exercise to evaluate the use of postal service locations as testing sites in order to increase the coverage and accessibility of COVID-19 testing, particularly for individuals residing remotely or within communities that are deemed to be at risk. | Modeling study | The authors evaluated an alternative testing location option (postal services) to address the issue of limited access to testing for a subset of the population. | The authors evaluated an alternative logistical network to administering COVID-19 testing, chiefly the possibility of utilizing the resources of the USPS. Through their modeling, they found that incorporating USPS services would allow for greater accessibility to testing of the population through on-site testing or the delivery of testing kits to homes. |
| Sotgiu & Dobler  2020 | European Respiratory Journal | Global | Academic publication | The describe the impact of social stigma on health-related behaviours related to the COVID-19 pandemic. | Commentary | Social stigma of being positive for corona virus inhibits people from getting tested because it may reveal they did not abide by social rules (PPE, isolation, etc.) and be shamed. This creates anxiety and uncertainty in people who don't want to be known for spreading the virus.  Communication: The infodemic of misinformation, news, mixing facts, and rumours leads people to make poor health-related behaviours, including testing. | Education about symptoms, testing, outcomes, etc.  Honest, direct, simple communication from leaders about testing. |
| Thappa 2002 | International Journal of Health Sciences and Research | India | Academic publication | This correspondence article aims to summarize the reasons for testing hesitancy in India. | Text and opinion | A variety of barriers are reported.  (1) People with limited incomes are fearful of losing their jobs. (2) Waiting for test results (3) Living conditions for quarantine centre are suboptimal (consequences of testing positive): These centres are described as having poor lodging and food facilities, so people are reluctant to get tests. (4) Dilution of fear/threat of virus: testing hesitation turning into "ignorance". (5) COVID-19 fatigue: sense of fatigue among citizens of India owing to the fact there is an absence of any specific treatment for COVID-19 ("testing is done in vain"), that it is a disease that is much less harmful to young, healthy populations, and that there is a low frailty rate. (6) mental strain from lockdown and threat of being in quarantine; (7) history of hesitancy for new tests and vaccine amongst the population in India. | Targeted, specific information and education materials to address the lax attitude toward testing.  Regular updating of information and content. |
| The Unity Council  2020 | NGO website | United States | Grey literature | Reporting on Community testing program in Fruitvale Sanado Juntos Community (California).  This is a 2 page brief about the outcomes of the free testing event. It identifies three strategies that can improve testing in Latinx communities. | Brief report. | Access to testing | More COVID-19 testing and contact tracing to identify cases and provide resources to households.  Targeted public health outreach to marginalized communities.  Economic relief (food and housing assistance, income replacement for workers who need to quarantine).  Strategies were suggested but not implemented. |
| Thunstrom  2020 | Behavioural Public Policy | United States | Academic publication | To evaluate whether individuals are likely to test for COVID-19 infection, taking into account their financial abilities and willingness to pay, as well as their personality characteristics.  The authors used hypothetical scenarios to test the willingness of the participants to get tested. | Hypothetical field experiment (simulated RCT, between- subjects design)  (n=1000 total of whom n=890 observations were included due to not having already been tested for COVID-19 and having complete data) |  |  |
| Tower Hamlets Council | Government website | UK | Media | Community engagement for Test and Trace | Media | None specifically identified. | Strong network of community partners including community navigators who work one-to-one with residents to help them identify an access the support they need to enhance health and wellbeing. They gather insights on barriers to engagement, identifying support needs and then meeting these needs.  Working with other council services (eg sexual health commissioned services) and partners to strengthen the support offer within community, voluntary and faith sector groups, and enable identification of extra support the council may need to provide to increase uptake and engagement among these communities. This may include, for example, bespoke communications for specific communities, community champions, and proactive/reactive support for self-isolation. |
| UNICEF  2020 | Governmentwebsite | Global | White paper | To describe social stigma related to COVID-19, its negative effects and how to address it. | Guidance document | Social stigma can inhibit people from getting tested by having them hide their illness, stop healthy behaviour, and discourage them from seeking help. | Communication: words matter, become literate to effects of stigma.  Governments should do their part to influence positive behaviours by spreading facts, engaging influencers, amplifying voices of affects, portray different ethnic groups, and act ethically.  Address the infodemic by clearing up misinformation as soon as it arises, correct misconceptions, have one clear voice and spread facts. |
| Zimba | medRxiv | United States | Preprint | To determine important drivers of decisions to obtain a SARS-CoV-2 test in the context of increasing community transmission.  Survey to assess the relative importance of type of SARS-CoV-2 test, specimen type, testing venue, and results turnaround time. | Discrete choice experiment  (n=5098 eligible from a current cohort study; n=4793 participated) | Findings suggest NP swabs may be a deterrent to testing. | Participants identified that rapid receipt of results, tests that detected current and past infections, cheek swabs and at-home self-collection were the most preferable testing service attributes.  Simulating changes in attribute trade-offs individually, providing immediate or same day test results, providing both PCR and serology, or collecting oral specimens would increase testing uptake the most. |
